# The Brazilian collaborative on antimicrobial stewardship: A value-based healthcare approach

**DOI:** 10.1016/j.bjid.2024.103738

**Published:** 2024-04-25

**Authors:** Ruan de Andrade Fernandes, Marcelo Carneiro, Marcia Makdisse, Natanael Sutikno Adiwardana, João Paulo Telles, Claudia Fernanda de Lacerda Vidal, André Luís Franco Cotia, Muir Gray, The Brazilian Collaborative on Antimicrobial Stewardship

**Affiliations:** a Rede D'Or São Luiz, São Paulo, SP, Brazil; b Universidade de Santa Cruz do Sul, Rio Grande do Sul, RS, Brazil; c Academia VBHC, São Paulo, SP, Brazil; d A.C. Camargo Câncer Center, São Paulo, SP, Brazil; e Universidade Federal de Pernambuco, Pernambuco, PE, Brazil; f Hospital Israelita Albert Einstein, São Paulo, SP, Brazil; g Oxford Value and Stewardship Programme (OVSP) & Academia VBHC, Oxford, UK

**Keywords:** Value based health care, Infectious diseases, Antimicrobials, Clinical outcomes

Dear Editor,

The misuse of antibiotics and its consequence of Antimicrobial Resistance (AMR) are critical for health systems in low- and middle-income countries, where health resources are scarce, and inequalities in access to diagnostic and therapeutic options are even greater.^1^ A study in 9 Latin American countries showed a significant improvement in antimicrobial use when more comprehensive Antimicrobial Stewardship Programs were available.^2^ Furthermore, prevention of antimicrobial drugs-related toxicity should also be a potential benefit of ASP, mainly with vancomycin, aminoglycosides and voriconazole.^3^ However, despite these data, the first nationwide survey involving 957 Brazilian Adult ICUs found that 52.5 % of these hospitals still do not have ASPs in ICUs.^4^ In response to these challenges, a task force was created in Brazil to promote ASPs. The initiative is a national collaboration including infectious disease specialists, microbiologists, pharmacists, and specialists in Value-Based Healthcare (VBHC) to promote the responsible use of antimicrobial agents. Our collaborative is committed to the principles of VBHC,^5^ a concept of four value-pillars: (i) Technical Value (i.e., best possible outcomes with available resources, and expressed in Porter's Value Equation, (ii) Personal Value, (i.e., appropriate care and how well the outcomes achieved relates to the goals of individuals); (iii) Allocative value (i.e., equitable resource distribution), (iv) Societal Value (i.e., contribution of healthcare to society as a whole). This broader concept of VBHC is of utmost importance in countries, such as Brazil, to reduce health inequities. The Brazilian Collaborative on Antimicrobial Stewardship unifies the debate across different medical societies and key professionals on the topic of antimicrobial management in Brazil, while it leverages the reach of actions in relation to what members could achieve individually. In conclusion, we hope that our efforts will inspire other low- and middle-income countries that seek to follow and contribute to the global effort of ASP-VBHC implementation, and to the reduction of AMR and the environmental tragedy that is unfolding. Our goal with this learning collaborative is to offer comprehensive and safe care through continuous value improvement and the adoption of a culture of stewardship.

## Conflicts of interest

The authors declare no conflicts of interest.

## References

[1] Mendelson M., Røttingen J.A., Gopinathan U. (2016). Maximising access to achieve appropriate human antimicrobial use in low-income and middle-income countries. Lancet.

[2] Quirós R.E., Bardossy A.C., Angeleri P. (2022). PROA-LATAM Project Group. Antimicrobial stewardship programs in adult intensive care units in Latin America: implementation, assessments, and impact on outcomes. Infect Control Hosp Epidemiol.

[3] Telles J.P., Morales R., Yamada C.H. (2023). Optimization of antimicrobial stewardship programs using therapeutic drug monitoring and pharmacokinetics-pharmacodynamics protocols: a cost-benefit review. Ther Drug Monit.

[4] Menezes R.M., Gonçalves M.R.S., Costa M.M.M. (2022). Antimicrobial Stewardship Programmes in Brazil: introductory analysis. Res, Soc Dev.

[5] European Commission. Defining value in “value based healthcare”. Report of the Expert Panel on effective ways of investing in Health (EXPH). Luxembourg: Publications Office of the European Union, 2019. [cited 30 October 2019]. Available at: https://ec.europa.eu/health/publications/defining-value-value-based-healthcare-0_en. [Accessed on 10 June 2023].

